# Limitations to Reproductive Success in the Dioecious Tree *Rhamnus davurica*

**DOI:** 10.1371/journal.pone.0081140

**Published:** 2013-12-06

**Authors:** Juan Wang, Chunyu Zhang, Xiuhai Zhao, Klaus V. Gadow

**Affiliations:** 1 Key Laboratory for Forest Resources and Ecosystem Processes of Beijing, Beijing Forestry University, Beijing, China; 2 Faculty of Forestry and Forest Ecology, Georg-August-University Göttingen, Büsgenweg 5, Göttingen, Germany; United States Department of Agriculture, United States of America

## Abstract

The reproductive success of a female plant in a dioecious species may be affected by pollen limitation and resource limitation. This study presents evidence that the reproductive success of the dioecious understorey tree species, *Rhamnus davurica*, is affected by the distance to the nearest male. The sex ratios were female-biased, although showing fluctuations in the three years of conducting the study. The mortality rate of females was higher than that of males indicating a trade-off between reproduction and survival. Altogether 49 females, designated as “focal females”, were randomly selected for monitoring their reproductive status between April and October in 2010. But successful reproduction (meaning that the flowering female trees had fruit in the fruiting season) was observed only in 28 females in 2011 and 16 females in 2012. The method of path analysis was applied to determine the effect of topography, local competition and proximity to the nearest male on the fruit set of the females. In the three years of the study, elevation, competition and female size had no significant effect on the fruit set. The distance to the nearest male, however, had a significant effect on fruit set. Number of fruits and fruit set were decreased with increasing distance to the nearest male. It was possible to estimate maximum fruit set, based on the comparatively large dataset. The number of fruits and the fruit set are exponentially related to the distance to the nearest male and the relationships are described by an exponential model. The results of this study support the importance of pollen limitation on the reproductive success in *Rhamnus davurica*.

## Introduction

The reproductive success of plants depends, among other things, on the number of flowers and their fertilized proportion which is known as the “fruit set”. Fruit set is a complex process which depends on pollen quantity [1], pollen quality [2] and resource limitation (which in this particular case means soil nutrients) [3], etc. Pollen limitation in wind-pollinated plants occurs when a plant receives inadequate quantity of pollen or low-quality pollen. Although wind pollination plants have a higher transfer efficiency than animal-pollinated plants, numerous empirical studies reported significant pollen limitations and under specific environmental conditions [4]–[5]. Pollen limitation of fruit set is different among different species [6]. The fruit set may increase with increasing flowering plant density, as reported, for example by Crone and Lesica [7], also by Rapp et al [8]. While a negative correlation between fruit set and floral density in *Taxus* [9]. Recently *L. rubrum* was found that there was no correlation between the fecundity and the floral density [10]. Resource limitation may be caused by topography, neighboring competition and high plant densities.

This study mainly deals with the pollen limitation and the female reproductive success in the dioecious small tree *Rhamnus davurica*. *Rhamnus davurica* occurs in broadleaved and conifer mixed forests in Northern China and Eastern Russia. It is a wind-pollinated small tree in our plot. Obviously, we may assume that the pollen environment is strongly influenced by the proximity and density of male neighbors. Female flowers in the vicinity of males will receive larger quantity of pollen than females which are located at greater distances from a male. Thus, we may assume that differences in the pollination environment, i.e. differences in the proximity of males within the local neighborhood range, may be responsible for differences in female fruit setting.

A lot of researches found a positive correlation between the reproductive success and pollen limitation [8], [11]–[14]. As far as we know, this study is the first one which investigate the correlation between fruit set and pollen limitation in dioecious shrub *Rhamnus davurica*. We try to demonstrate that the distance to the nearest *R. davurica* male has an important influence on the reproductive success, i.e. on the number of fruits as well as the fruit set of females. The main objectives of this study are as follows: 1) What is the correlation between the pollen limitation and female fruit set in a dioecious species of *R. davurica*? 2) What is a general conclusion by modeling the relation between fruit set and some influencing factors (including topography, pollination environment, neighboring competition and tree size)?

## Materials and Methods

### Ethics Statement

All necessary permits were obtained for the field studies. The study was approved by the Ethics Committee of *Jiaohe* Administration Bureau of the Forest Experimental Zone in Jilin province, in Northeastern China.

### Study Area

The research was performed at the *Jiaohe* experimental forest in Jilin province, in northeastern China (43°58′N, 127°43′E; elevation 450 m a.s.l.). The average annual temperature is 3.8°C. The hottest month is July with a mean daily temperature of 21.7°C, while the coldest month is January with an average daily temperature of −18.6°C. The mean annual precipitation accounts for 695.9 mm. The experimental area is different-aged broad-leaved and coniferous mixed forest. The oldest tree is about 150 years old. The main coniferous species are *Abies holophylla* and *Pinus koraiensis* while the dominant deciduous species are *Fraxinus mandshurica*, *Tilia amurensis* and *Acer mono*.

The species of interest in this study is *Rhamnus davurica* which occurs as a shrub or small tree in the broadleaved/coniferous forest of Eastern Russia and Northern China. The species is common at the study site. Most individuals have a single trunk, but few develop double or multiple trunks. The trees start flowering when the DBH is about 2 cm. The DBH of *R. davurica* is not greater than 12 cm in our research site. The sex ratio is the sex ratio of flowering plants in each of three years. Both genders of *R. davurica* produce flowers in May and are primarily pollinated by wind. The fruits ripen from August to September are 5–7 mm in diameter while the weight ranges between 0.010 and 0.049 g. Their drupaceous fruits change to purple and black when they ripen. The fruits with seeds are mainly autochorously dispersed. Wind dispersal over long distances has not been observed [15]. 2–3 seeds are found within a fruit, and no empty seeds are reported.

**Figure 1:**
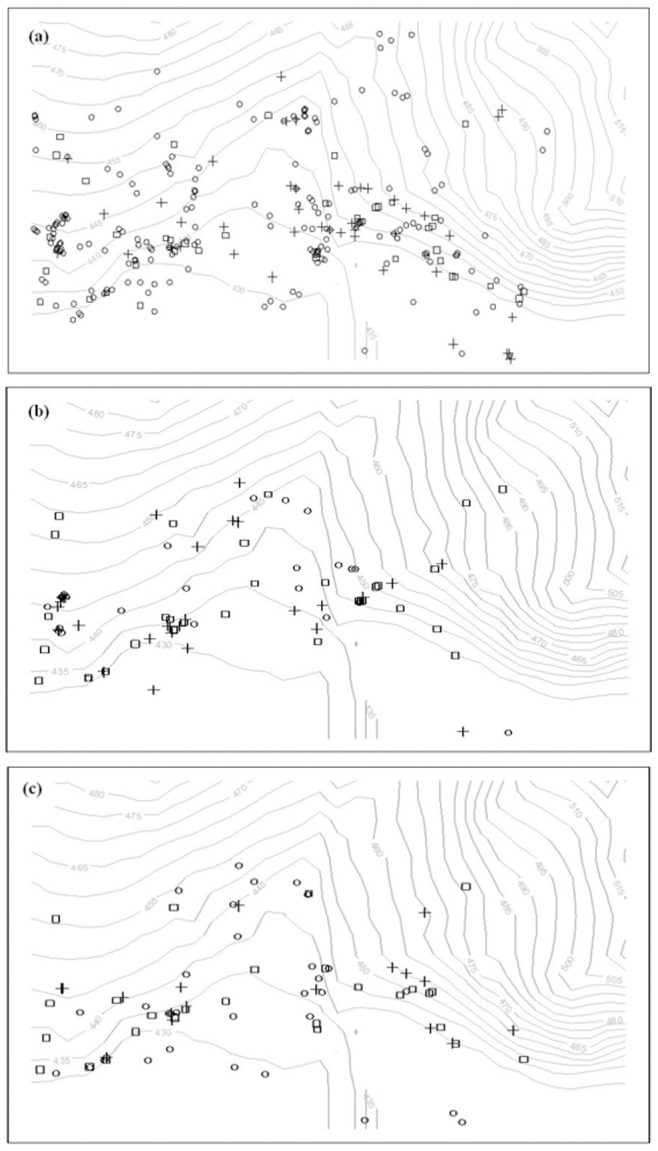
Spatial distribution of *R. davurica* in a 360×660 m research plot in *Jiaohe* experimental forest in northeastern China in three years. (a) indicates the distribution in 2010; (b) indicates the distribution in 2011; (c) indicates the distribution in 2012; Squares indicate *Rhamnus davurica* males; circles indicate females not studied; crosses show females selected for this study. Gray lines show the elevation contours at 5-m intervals.

The field plot, which covers a rectangular area of 23.76 ha (660×360 m), was subdivided into 594 square cells, each covering an area of 400 m^2^ (20×20 m). There are altogether 588 *R. davurica* trees in the experiment plot. For all the *R. davurica* trees the reproductive status and mortality were investigated in May in the three consecutive years.304 females and 54 males were flowering in 2010, 53 females and 37 males were flowering in 2011, 54 females and 34 males were flowering in 2012. Not all the *R.davurica* trees were suitable for doing our experiment because of their different shapes. Some trees which had a short trunk and a big crown were easy to use. So we randomly chose female trees which were flowering, fruiting and suitable as “focal females”. Altogether 49 females were randomly selected for monitoring their reproduction status between April and October 2010. 28 females were selected in 2011 and 16 females in 2012. Some selected female trees produced flowers and fruits continuously in three years; some selected female trees just showed reproductive behavior in one or two of the three years. The locations of the male and female trees are shown in Figure 1. The size structure of these sample trees from 2010 to 2012 year is shown in Table 1.

**Table 1:** The size structure of the selected Rhamnus davurica females and all flowering males in the analysis.

Year	Sex	DBH classes (cm)					Height classes (m)				
		1–2	2–4	4–6	6–8	8–10	1–2	2–4	4–6	6–8	>8
2010	Female	1	15	12	10	11	0	13	22	10	4
	Male	2	10	5	3	0	1	9	6	4	0
2011	Female	2	9	11	3	3	3	10	10	5	0
	Male	0	12	10	5	0	2	10	12	3	0
2012	Female	0	7	5	2	2	0	6	9	0	1
	Male	0	12	7	2	0	0	13	6	2	0

### Topographical Variables

The intersections of the grid lines are called “nodes” in the experiment plot. The relative height differences between adjacent nodes were measured. The elevation of the starting node was assessed using the GPS device (*Magellan eXplorist 600*). Thus it was possible to calculate the elevation of all the nodes. The elevation of a particular cell was calculated as the mean value of the elevations of its four corner nodes.

The cell slope was estimated as the mean angular deviation from the horizontal plane of each of the four triangular planes which were formed by connecting three of its adjacent corners [16]. Slope is a circular variable, and sin(slope) was computed in order to use slope in linear models. The convexity of a cell was calculated as the elevation of the focal cell minus the mean elevation of the eight surrounding cells [17]. For the edge cells, convexity was taken as the elevation of the center point minus the mean of the four corners. Positive and negative convexity values respectively indicate convex (ridge) and concave (valley) land surfaces.

The aspect of a cell can be obtained from the average angle of the four triangular planes that deviate from the north direction. Aspect is also a circular variable, and sin(aspect-50) was calculated in order to use aspect in linear models. This turns aspect into an index which varies from −1 to 1.

### Field Observations

The diameter at breast height (DBH) and tree height of each of the focal females was measured. Only younger branches can bear flowers and fruits for female *R. davurica* [15]. Ten chosen reproductive branches in eight crown compartments (four horizontal sections and two vertical layers) were tagged for each focal female during May 2010. The number of flowers and fruits were counted on all the selected reproductive branches in May and September, respectively. The reproductive branch sample selected for the counts was considered to be representative for the particular female. We counted the total number of reproductive branches per female and calculated the average flower/fruit number per reproductive branch based on the number of flowers/fruits for 10 representative branches per female. The total number of flowers/fruits was estimated by multiplying the averages flower/fruit number per reproductive branch with the total number of reproductive branches. Then the fruit set (R_fruitset_) of the focal female was calculated as follows: R_fruitset_ = (number of fruits/number of flowers). This variable may assume values between 0 and 1.

### Statistical Analysis

Two statistical methods were applied in this study. The first method involves the use of Pearson’s product moment correlation. The purpose of the Pearson’s correlation was to determine which of the topographic variables significantly correlated with fruit set, so as to then only include those variables in the path analysis. And four topographic variables elevation, terrain convexity, slope, aspect as described above were included in the Pearson’s correlation analysis.

The second method involves path analysis, which was first proposed by Wright [18]–[19], and is used to analyze systems of multiple causality. It represents a useful extension of the classical regression approach and can be applied to test multiple factors that may affect specific natural phenomena. In this study, the fruit set was used as response variable, with elevation, slope and aspect as predictor variables. Neighboring competition, pollination environment and tree size were also used as predictor variables. Competition from neighboring trees was computed using the Iterative Hegyi index. The Iterative Hegyi index was calculated in three stages: determine the dynamic of radius of the competition zone; select active competitors within competition zone; calculate the index. The more details about the Iterative Hegyi index was supplied by Lee et at. [20]. To illustrate the effect of neighboring trees on the focal females, the neighborhood radius of the competition zone was defined to be 4 m. The distance (*d_1_*) between a specific focal female and its nearest male neighbor was measured to assess the pollination environment for each focal female. The tree size of a female was measured by diameter at breast height.

**Figure 2:**
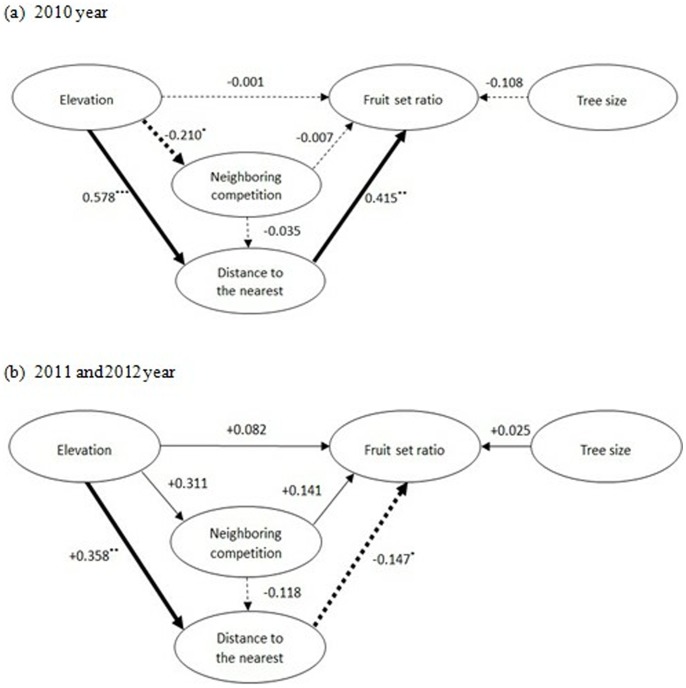
Path diagramms showing the influence of elevation, local competition from neighboring trees, pollination environment and tree size and on the fruit set of *R. davurica*. The arrows define the cause-and-effect relationships in the path diagram for the causal model. Path coefficients are given for each arrow. Female size is expressed as stem diameter (cm) at breast height measured in May 2010. ***p<0.001, **p<0.01, *p<0.05.

The path analysis expresses more than the strength of the linear relationship between two variables. The causal diagram in Figure 2 comprises the set of the selected variables. Each variable is connected by an arrow to another variable with which it has a causal influence, the arrowhead indicating the direction of the causal relationship. The number of observed females only accounts for 28 females in 2011 and 16 females in 2012. It can’t meet the requirement of sample size in path analysis. Thus, the data of observational females in 2011 and 2012 year were combined to calculate the causality. Finally, we obtained two causal diagrams (one is for 2010 and the other one is for 2011 and 2012 year). Path analyses were conducted using the “*sem*” package of the *R* software. We also estimated maximum fruit set for different distances to the nearest male, using linear quantile regression implemented in the package *quantreg* of *R* software [21].

## Results

### Sex Ratio

The sex ratio of flowering plants in *R. davurica* was significantly female-biased in 2010 and 2012, but was 1∶1 in 2011. The sex ratios in the different *DBH* classes were consistent with the population sex ratios, except in the 1–2 cm *DBH* class in 2010 and in the 1–2 cm, 2–4 cm, 4–6 cm *DBH* classes in 2012. There were no flowering trees in >8 cm DBH class in 2011 (Table 2).

**Table 2:** Number of stems and sex ratios of *Rhamnus davurica* in different DBH classes in the 360×660 m research plot, G(P) = G-test results.

		DBH classes (cm)					Total
		1–2	2–4	4–6	6–8	>8	
2010	Male	3	22	18	11	0	54
	Female	9	78	136	62	19	304
	None	11	88	87	28	10	224
	Male/Female	0.33	0.28	0.13	0.18	0	0.18
	G(P)	3.01^ns^	33.08***	102.07***	39.04***	25.66***	192.33***
2011	Male	1	16	14	6	0	37
	Female	3	18	21	11	0	53
	None	13	131	182	78	28	432
	Male/Female	0.33	0.89	0.67	0.55	–	0.7
	G(P)	0.93^ns^	0.12^ns^	1.39^ns^	1.45^ns^	–	2.84^ns^
2012	Male	2	16	10	6	0	34
	Female	0	17	19	15	3	54
	None	3	93	98	38	11	243
	Male/Female	–	0.94	0.53	0.40	0.00	0.63
	G(P)	2.77^ns^	0.03^ns^	2.84^ns^	3.98*	4.16*	4.59*

Most of the selected females were distributed in the 2–4 cm, 4–6 vcm, 6–8 cm and 8–10 cm DBH classes in 2010. But most of the selected females were distributed in the 2–4 cm and 4–6 cm DBH classes in 2011 and 2012 (Table 1). Most flowering males were also distributed in the smaller DBH and height classes.

**Figure 3:**
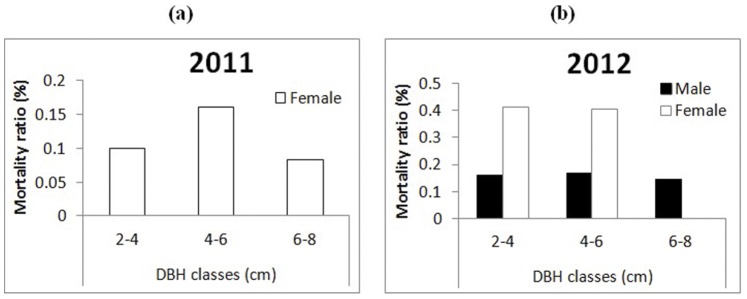
The mortality ratio for males and females in different DBH classes in2011 and 2012.

The female mortality rates were higher than those of males in each DBH class in 2011 and 2012 (Fig. 3). The male mortality rate was zero in each DBH class in 2011, but nearly 20% in each DBH class in 2012. The female mortality rate was higher in the 4–6 cm DBH class than in the 2–4 cm and 6–8 cm DBH classes in 2011(Fig. 3).

### Distance to the Nearest Male and Reproductive Success

**Figure 4:**
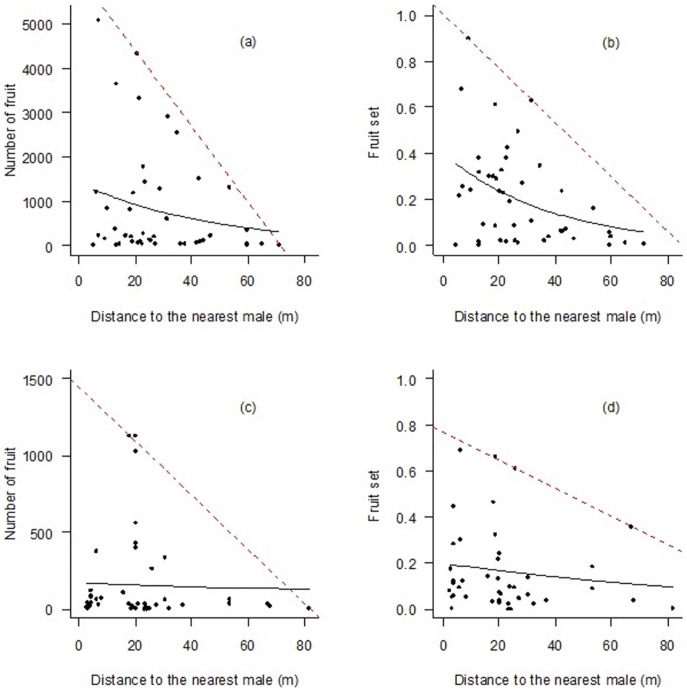
Relationships between female reproductive success (fruit number and fruit set) and the distance to the nearest male for *Rhamnus davurica*. (a) and (b) calculated from observational data in 2010 year; (c) and (d) calculated from observational data in 2011 and 2012 years. The solid lines represent the exponential regression of reproductive success (fruit number and fruit set) as a function of the distance to the nearest male. Dashed lines represent maximum fruit set, evaluated by quantile regression with tau = 0.975 percentile.

Figure 4 presents the relationships between the reproductive success (which includes number of fruits and fruit set) and the distance to the nearest male. The number of fruits and the fruit set are exponentially related to the distance to the nearest male (Table 3). Females in closer proximity to males had a greater chance of reproductive success than females further away from males. The number of fruits was not significantly correlated with the distance to the nearest male (*r* = −0.2282, *p* = 0.13), whereas the fruit set was (*r* = −0.4174, *p*<0.01). It decreased with the increasing distance to the nearest male. The distance at which fruit set was more than 50% of its maximum value was 31.6 m. Females in closer proximity to males had higher chance to the reproductive success than females further away from males.

**Table 3:** The exponential relationships between the reproductive success (including number of fruits and fruit set) and the distance to the nearest male (*d*_1_).

Year	Number of fruits	Fruit set
2010	*N*_fruit_ = 1408.4×e^−0.0211·*d1*^	*R*_fruitset_ = 0.404×e^−0.0268·*d1*^
2011 and 2012	*N*_fruit_ = 171.53×e^−0.003968·*d1*^	*R*_fruitset_ = 0. 1978×e^−0.008752·*d1*^

Based on these observations, the maximum fruit set for a given distance to the nearest male was estimated by using quantile regression. The linear relationship between fruit set and distance to the nearest male for the 0.975 quantile in 2010 year is *R_fruitset_* = 1.008-0.0119 (*Distance*, m). And the relationship in 2011 and 2012 years is *R_fruitset_* = 0.766-0.00613 (*Distance*, m).

### Path Analysis

Based on the Pearson’s correlation results, the fruit set was negatively associated with the elevation in *R. davurica* (correlation coefficient *r* = −0.26, *p*<0.03), and neutrally with other factors (convexity, slope and aspect; *p*>0.05) in 2010. While no significant association between the fruit set and topography was found both in 2011 and 2012.

A large proportion of females produced no fruits, and fruit set was only 23.4%, 16.5% and 19.8% in 2010, 2011 and 2012, respectively, indicating a low reproductive efficiency. As expected, the number of fruits in the female trees was positively related to the number of their flowers (Pearson’s correlation, 2010: *r* = 0.70, *p*<0.0001; 2011: *r* = 0.89, *p*<0.0001; 2012: *r* = 0.97, *p*<0.0001).

There was a wide range of distances to the nearest male tree due to the spatial variation in male density. The mean value of the distances to the nearest male was 19.95 m (SE = 2.28, range = 4.91∼71.33) in 2010, 22.95 m (SE = 4.42, range = 2.91∼81.79) in 2011, and 26.13 (SE = 5.26, range = 2.55∼84.79) in 2012 for the focal females. More than 80% of the nearest male neighbors were located within 43 m of a female in the three observational years.

Elevation, female size, the distance to the nearest male and the neighboring competition were included in the path analysis. Elevation, competition and female size had no significant effect on fruit set for the species (Figure 2). The distance to the nearest male, however, had a negative effect on fruit set in the three observational years.

## Discussion

### Sex Ratio

The sex ratios of flowering plants in the entire population were female-biased, although fluctuating in the three continuous years of the study. Previous studies reported a female-biased sex ratio in moist environments [22]–[23]. Yu [24] found that the sex ratio of *Pistacia chinensis* was often male-biased in an environment with insufficient soil nitrogen. These results are congruent with the Batesman’s principle which suggests that female trees allocate higher reproductive costs when compared with males.

The sex ratios of the entire population were female-biased in 2010 and 2012, while the deviation from 1∶1 was not significant in 2011. These results may be ascribed to the Batesman’s principle which suggests that female trees allocate higher reproductive costs when compared with males. Male *R. davurica* trees merely produce flowers for pollen donation, whereas females need to produce flowers for the formation of fruits. Obviously, females allocate more resources to reproduction [25]–[26]. In order to compensate for the higher reproductive costs, females often reduce the frequency of reproduction [26]. The sex ratios in this study were not significantly different from 1∶1 in the smaller DBH classes in 2011 and 2012. Most of the females which had reproduced in 2010 did not contribute to reproduction in 2011 and 2012. Meanwhile, most of selected female trees did not distributed in bigger DBH classes (Table 1). This observation suggests that females are much more sensitive to the reproductive cost than males. After reproduction, females apparently need more time than males to balance the reproductive costs. Another relevant observation in this context is the fact that female mortality was much higher than male mortality in the periods 2010 to 2011 (no male mortality, Fig. 3) and 2011 to 2012 (in the smaller DBH classes).

The female mortalities were higher than male mortalities in three consecutive years. These results suggest that females allocate more resources to reproduction, which may affect their survival, suggesting a possible trade-off between reproduction and survival.

### Pollination Effect and Path Analysis

In a dioecious species, limited pollen receipt, which caused by greater distances between male and female plants, may limit reproductive success [27]. The study by Field et al. [28] reported that the range of pollen grains on stigmas significantly affected the percentage of seed set. However, the fruit set, representing the fertilized proportion of a dioecious species may be affected mainly by post-pollination processes [29]. The failure of some flowers to fruit may be due to the lack of pollination. Fruit set significantly decreased when the distance between females and their neighboring males was more than 27 m in the dioecious *Simmondsia chinensis* plant [30]. In this study, more than 80% of the nearest male neighbors were located within 43 m of a female. In a study by de Jong et al. [31], seed set was found to decrease significantly with increasing distance to the nearest male in three dioecious species (*Salix repens*, *Asparagus offıcinale* and *Bryonia dioica*) due to pollen limitation.

The relationship between the fruit set and the distance to the nearest male was described by a quadratic function in *Diospyros Montana* [32]. de Jong et al. [31] report that seed set declined with distance in meters to the nearest male in four dioecious species. But an exponential model was used in this study to describe the relationships between fruit set and distance to the nearest male. The estimates of a theoretical maximum fruit set are an important element of life-history theory. However, this study shows that the potential female reproductive success is limited by unknown factors, possibly including density-dependent pollen movement.

Our results show that the fruit set decreased with increasing distance to the nearest *R. davurica* male. Assuming equal pollen quality and quantity in all the males, and homogeneous spatial patterns, we may assume that distance is the most important factor limiting pollen transfer. Other studies reported similar findings. For example, Somanathan and Borges [33] found that fruit set was strongly and negatively correlated with distance to the nearest male in the dioecious *Diospyros montana*. In a study involving the three tropical dioecious species *Neolitsea dealbata*, *Litsea leefeana* and *Diospyros pentamera*, House [34] found that the fruit set in females was negatively related to the distance to the nearest flowering male. However, other studies report for example neutral effects. The distance between a female and the nearest male had no effect on the fruit set in the dioecious *Gardenia actinocarpa* [35].

Both sexes seem to have similar habitat preferences which will influence the pollen environment around the focal females (Fig. 1). The fruit set of a plant depends on successful pollination, but local environment and surrounding competition may also be important. According to Gulias and Traveset, the change of habitat traits may influence fruit set. *Rhamnus lycioides* had a higher fruit set at the mountain than at the coast [36]. In this study, however, topography and neighboring competition had no significant effect on the fruit set.

House [34] examined the effect of male tree density on the fruit set in females for three dioecious tree species in tropical, north-eastern Australia. He found that fruit set was not correlated with female size. Contrary to this observation, Berry and Gorchov [37] reported that the number of flowers and fruits borne on a female were dependent on female size. In this study, the female size had no significant effect on fruit set.

The Pearson’s correlation and the path analysis had different results with regard to the impact of elevation on fruit set. Pearson’s correlation is a regression model, but path analysis is a determined model. The fruit set was negatively associated with the elevation based on the Pearson’s correlation results. This indicates that fruit set significantly decreases with increasing elevation. Path analysis examines the cause-and-effect relationships in path diagram. According to the path coefficient, the elevation is not the direct cause of the variation of fruit set. These results may indicate that the number of male trees were less than female in higher elevation. The fruit set was negatively correlated with the distance of the nearest male tree in our study. The fruit set decreases with increasing elevation because of short of male trees.
